# Obesity and kidney disease: Hidden consequences of the epidemic

**DOI:** 10.4102/phcfm.v9i1.1435

**Published:** 2017-10-25

**Authors:** Csaba P. Kovesdy, Susan L. Furth, Carmine Zoccali

**Affiliations:** 1 Division of Nephrology, Department of Medicine, University of Tennessee Health Science Center, United States; 2 Nephrology Section, Memphis VA Medical Center, United States; 3 Department of Pediatrics, Perelman School of Medicine, University of Pennsylvania, United States; 4 CNR - IFC Clinical Epidemiology and Pathophysiology of Renal Diseases and Hypertension, Reggio Calabria, Italy

## Introduction

In 2014, over 600 million adults worldwide were obese. Obesity increases the risk of developing major risk factors for chronic kidney disease (CKD), like diabetes and hypertension, and it has a direct impact on the development of CKD and end-stage renal disease (ESRD). The good news is that obesity is largely preventable. Education and awareness of the risks of obesity and a healthy lifestyle, including proper nutrition and exercise, can dramatically help in preventing obesity and kidney disease. This article reviews the association of obesity with kidney disease on the occasion of the 2017 World Kidney Day.

## Association of obesity with chronic kidney disease and other renal complications

Numerous studies have shown an association between measures of obesity and both the development and the progression of CKD. In general, the associations between obesity and poorer renal outcomes persist even after adjustments for possible mediators of obesity’s cardiovascular and metabolic effects, suggesting that obesity may affect kidney function through mechanisms in part unrelated to these complications. The deleterious effect of obesity on the kidneys extends to other complications such as nephrolithiasis and kidney malignancies.

## Mechanisms of action underlying the renal effects of obesity

**FIGURE 1:**
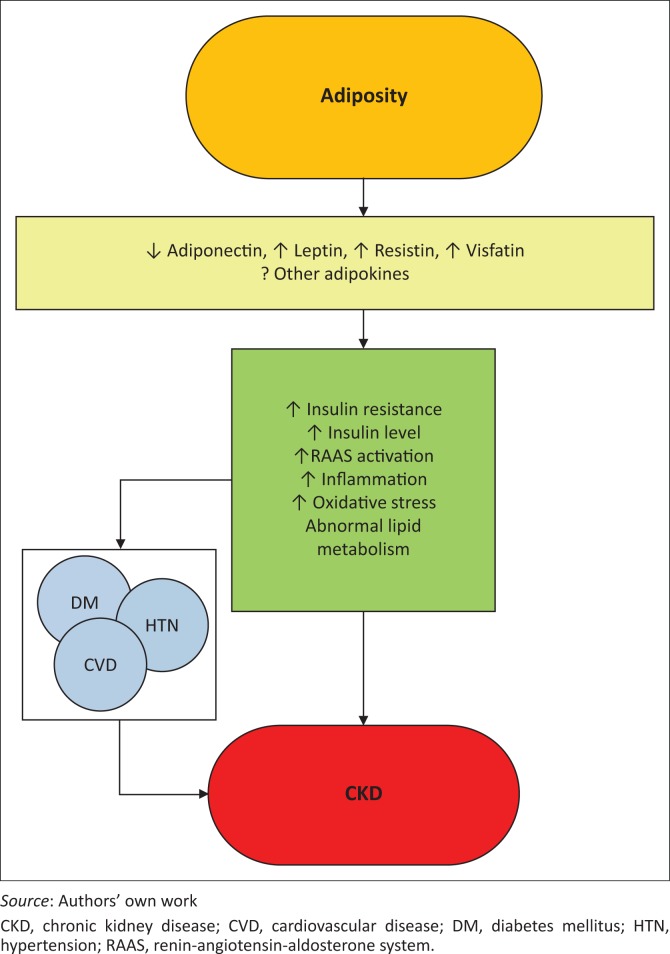
Putative mechanisms of action whereby obesity causes chronic kidney disease.

The exact mechanisms whereby obesity may worsen or cause CKD remain unclear. Some of the deleterious renal consequences of obesity may be mediated by comorbid conditions such as diabetes mellitus or hypertension, but there are also effects of adiposity which impact the kidneys directly via production of (among others) adiponectin, leptin and resistin (Figure 1). These include the development of inflammation, oxidative stress, abnormal lipid metabolism, activation of the renin–angiotensin–aldosterone system and increased production of insulin and insulin resistance.^1^

**FIGURE 2:**
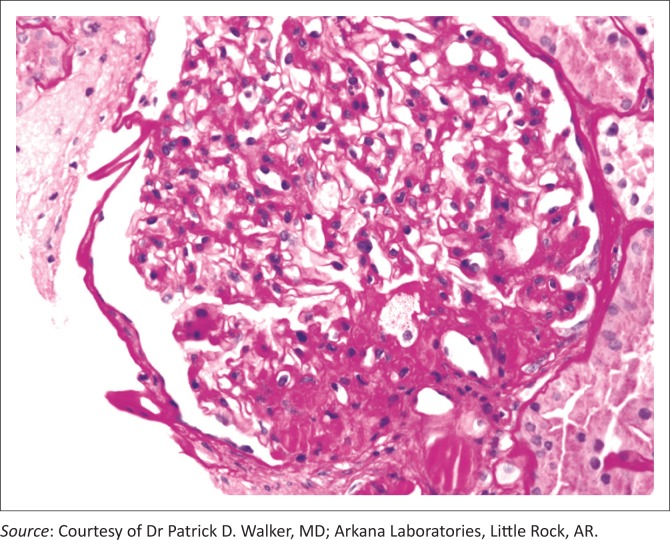
Obesity-related perihilar focal segmental glomerulosclerosis on a background of glomerulomegaly. Periodic acid-Schiff stain, original magnification 400x.

These various effects result in pathologic changes in the kidneys including ectopic lipid accumulation and increased deposition of renal sinus fat, glomerular hypertension and increased glomerular permeability, and ultimately the development of glomerulomegaly, and focal or segmental glomerulosclerosis (Figure 2).^2^ The incidence of the so-called obesity-related glomerulopathy has increased 10-fold between 1986 and 2000.

Obesity is associated with a number of risk factors contributing to nephrolithiasis, such as lower urine pH and increased urinary oxalate, uric acid, sodium and phosphate excretion. The insulin resistance characteristic of obesity may also predispose to nephrolithiasis through its impact on tubular Na–H exchanger and ammoniagenesis, and the promotion of an acidic milieu.^3^

The putative mechanisms behind the increased risk of kidney cancers observed in obese individuals include insulin resistance, chronic hyperinsulinemia and increased production of insulin-like growth factor 1, which may exert stimulating effects on the growth of various types of tumour cells. More recently, the endocrine functions of adipose tissue, its effects on immunity and the generation of an inflammatory milieu with complex effects on cancers have emerged as additional explanations.^4^

## Obesity in patients with advanced kidney disease: The need for a nuanced approach

In a seemingly counterintuitive manner, obesity has been consistently associated with lower mortality rates in patients with CKD and ESRD.^5^ It is possible that the seemingly protective effect of a high body mass index (BMI) is the result of the imperfection of BMI as a measure of obesity. However, there is also evidence to suggest that higher adiposity, especially subcutaneous (non-visceral) fat, may also be associated with better outcomes in ESRD patients. Such benefits may be present in patients who have very low short-term life expectancy, such as most ESRD patients, including among others benefits from better nutritional status.

## Potential interventions for management of obesity

### Countering chronic kidney disease at population level

In the United States, Healthy People 2020, a programme that sets 10-year health targets for health promotion and prevention goals, focuses on both CKD and obesity. A successful surveillance system for CKD has already been implemented in some places such as the United Kingdom,^6^ which may serve as a platform to improve the prevention of obesity-related CKD. Campaigns aiming at reducing the obesity burden are now at centre stage worldwide and are strongly recommended by the WHO, and it is expected that these campaigns will reduce the incidence of obesity-related complications, including CKD.

### Prevention of chronic kidney disease progression in obese people with chronic kidney disease

Obesity-related goals in obese CKD patients remain vaguely formulated, largely because of the paucity of high-level evidence intervention studies to modify obesity in CKD patients. In overweight or obese diabetic patients, a lifestyle intervention including caloric restriction and increased physical activity compared with a standard follow-up reduced the risk for incident CKD by 30%.^7^ In a recent meta-analysis collating experimental studies in obese CKD patients, interventions aimed at reducing body weight showed coherent reductions in blood pressure, glomerular hyperfiltration and proteinuria.^8^ Bariatric surgical intervention has been suggested for selected CKD and ESRD patients.

Globally, these experimental findings provide a proof of concept for the usefulness of weight reduction and angiotensin converting enzyme (ACE) inhibition interventions in the treatment of CKD in the obese. Studies showing a survival benefit of increased BMI in CKD patients, however, remain to be explained. These findings limit our ability to make strong recommendations about the usefulness and the safety of weight reduction among individuals with more advanced stages of CKD. Lifestyle recommendations to reduce body weight in obese people at risk for CKD and in those with early CKD appear justified, particularly recommendations for the control of diabetes and hypertension.

## Conclusions

The worldwide epidemic of obesity affects the earth’s population in many ways. Diseases of the kidneys, including CKD, nephrolithiasis and kidney cancers are among the more insidious effects of obesity, but which nonetheless have wide ranging deleterious consequences, ultimately leading to significant excess morbidity and mortality and excess costs to individuals and the entire society. Population-wide interventions to control obesity could have beneficial effects in preventing the development or delaying the progression of CKD. It is incumbent upon the entire health care community to devise long-ranging strategies towards improving the understanding of the links between obesity and kidney diseases, and to determine optimal strategies to stem the tide. The 2017 World Kidney Day is an important opportunity to increase education and awareness to that end.
